# Healthcare and Cancer Treatment Costs of Breast Screening Outcomes among Higher than Average Risk Women

**DOI:** 10.3390/curroncol30090620

**Published:** 2023-09-18

**Authors:** Nicole Mittmann, Kristina M. Blackmore, Soo Jin Seung, Christina Diong, Susan J. Done, Anna M. Chiarelli

**Affiliations:** 1 Department of Pharmacology & Toxicology, University of Toronto, 1 King’s College Circle, Toronto, ON M5S 1A8, Canada; 2 Sunnybrook Research Institute, Sunnybrook Health Sciences Centre, Toronto, ON M4G 3M5, Canada; 3 Ontario Health, 525 University Avenue, 5th Floor, Toronto, ON M5G 2L3, Canada; 4 HOPE Research Centre, Sunnybrook Research Institute, 2075 Bayview Avenue, Toronto, ON M4N 3M5, Canada; 5 ICES Central, 2075 Bayview Avenue, Toronto, ON M4N 3M5, Canada; 6 Laboratory Medicine Program, University Health Network, 200 Elizabeth Street, Toronto, ON M5G 2C4, Canada; 7 Dalla Lana School of Public Health, University of Toronto, 155 College Street, Toronto, ON M5T 3M7, Canada

**Keywords:** healthcare costs, false positive, breast cancer, digital mammography, family history, mammographic density, annual screening, biennial screening

## Abstract

Concurrent cohorts of 644,932 women aged 50–74 screened annually due to family history, dense breasts or biennially in the Ontario Breast Screening Program (OBSP) from 2011–2014 were linked to provincial administrative datasets to determine health system resource utilization and costs. Age-adjusted mean and median total healthcare costs (2018 CAD) and incremental cost differences were calculated by screening outcome and compared by recommendation using regression models. Healthcare costs were compared overall and 1 year after a false positive (n = 46,081) screening mammogram and 2 years after a breast cancer diagnosis (n = 6011). Mean overall healthcare costs by age were highest for those 60–74, particularly with annual screening for family/personal history (CAD 5425; 95% CI: 5308 to 5557) compared to biennial. Although the mean incremental cost difference was higher (23.4%) by CAD 10,235 (95% CI: 6141 to 14,329) per breast cancer for women screened annually for density ≥ 75% compared to biennially, the cost difference was 12.0% lower (−CAD 461; 95% CI: −777 to −114) per false positive result. In contrast, for women screened annually for family/personal history, the mean cost difference per false positive was 19.7% higher than for biennially (CAD 758; 95% CI: 404 to 1118); however, the cost difference per breast cancer was only slightly higher (2.5%) by CAD 1093 (95% CI: −1337 to CAD 3760). Understanding that associated costs of annual compared to biennial screening may balance out by age and outcome can assist decision-making regarding the use of limited healthcare resources.

## 1. Introduction

Breast cancer is a leading cause of morbidity and mortality for women in Canada, with a lifetime risk of 12.4% [1]. Canadian guidelines recommend mammography screening every two to three years for women aged 50–74 years [2]. Women at increased breast cancer risk may benefit from more frequent screening, leading to earlier detection and a reduced risk of interval cancers with poorer prognoses [3,4,5]. However, the benefits of tailored breast screening must be weighed against the possible harms of false positive results, such as unnecessary biopsies and diagnostic procedures that can cause anxiety and distress [6,7,8].

Generally, biennial screening has been shown to be more cost-effective than annual screening in terms of quality-adjusted life-years (QALYs) saved and lower incremental costs per life-year gained [9,10]. Higher incremental cost-effectiveness ratios (ICER) associated with annual screening were attributable to greater costs associated with an increased number of mammograms and evaluation of false positive results relative to life-years gained [9]. Conversely, a recent Canadian study found that although annual screening had higher ICERs, it was associated with greater life-years gained and QALY benefits [11].

Studies have also examined costs associated with screening based on breast cancer risk factors [12,13,14]. For women with a mammographic density ≥ 75%, annual versus biennial screening only had a 38% probability of being cost-effective at a willingness-to-pay threshold of CAD 100,000/QALY, with a mean ICER over CAD 500,000/QALY, exceeding the commonly cited cost-effectiveness threshold of CAD 50,000/QALY [12]. Similarly, annual mammography was not found to be cost-effective, regardless of age, breast density or family history, with ICERs of USD 340,000/QALYs or higher [13]. In contrast, a Spanish study found that annual screening for density > 50% and one or more risk factors (family history in first degree relatives, personal history of breast biopsy) was more cost-effective in terms of QALYs and had better harm–benefit ratios than biennial screening [14].

In the Ontario Breast Screening Program (OBSP) women aged 50–74 years are screened biennially; however, those with a family history of breast or ovarian cancer or personal history of ovarian cancer or density ≥ 75% are screened annually based on evidence that they are at higher-than-average risk of developing breast cancer [15,16,17,18,19,20]. Our recent studies found that mammography sensitivity was higher with annual screening for family/personal history compared to biennial and the risk of interval or higher stage invasive breast cancers reduced [21,22]. Annual screening for density ≥ 75% had equivalent sensitivity and similar risk of interval cancers, but lower specificity and higher abnormal recall and non-malignant biopsy rates [21,22].

While healthcare costs associated with screening based on risk factors are not fully known, these cohorts provide an opportunity to compare costs associated with annual screening for family/personal history or density ≥ 75% without family history to costs of biennial screening for those at average risk. This study compares mean and median health system resource utilization and costs (2018 CAD) and examines the incremental cost differences per woman between concurrent cohorts screened annually and biennially overall and associated with a false positive or breast cancer (screen-detected or interval) diagnosis. Although an earlier Canadian study examined mean healthcare costs per breast cancer case [23], to our knowledge, this is the first study to examine costs of breast cancers and false positive results associated with screening recommendations based on risk factors.

## 2. Materials and Methods

### 2.1. Study Population

The OBSP has operated since 1990 to deliver a population-based breast screening program to eligible women in a publicly funded health system [24]. Women are not eligible if they have a prior history of breast cancer or augmentation mammoplasty, currently have symptoms of breast disease or if screened in the High Risk OBSP. At OBSP centres, quality assurance on equipment exceeds that specified by the Canadian Association of Radiologist’s Mammography Accreditation Program (CAR-MAP) and radiologists and technologists are CAR-MAP accredited. During this study, women were screened at 162 OBSP centres. The study was approved by the University of Toronto Research Ethics Board and informed consent was not required.

This study employed a retrospective cohort design to identify concurrent groups of women 50–74 years of age screened in the OBSP, either annually for family/personal history, density ≥ 75% or biennially. Women were followed prospectively from their most recent screen between 1 January 2011 and 31 December 2014, until either a true negative, false positive or breast cancer diagnosis, with 31 December 2016 as the final date for outcomes. A complete description of the methods used to identify the cohort of screened women has been published [21,22].

### 2.2. Demographic and Risk Factor Information

Information for all women screened within the OBSP was obtained from data routinely collected by the Integrated Client Management System (ICMS). The technologist obtains relevant risk factor information from women at the screening visit. For family or personal history, data on female first-degree relatives with breast or ovarian cancer and age of diagnosis, as well as male first-degree relatives with breast cancer and personal history of ovarian cancer, were collected. Individual’s postal code of residence at screening was linked to the 2011 Canadian Census [25] to determine residence location and to assign area-based income quintiles. The presence of medical comorbidities was examined using the Charlson Comorbidity Index, a weighted score assigned to non-cancer comorbidities using hospitalization records in the two years preceding the index screening date [26].

### 2.3. Screening and Assessment Characteristics

Index screen date and age were based on the date of the most recent mammogram. The recommendation of the index screen was assigned based on risk factor information obtained at the previous screen (annual, density ≥ 75%; annual, family/personal history; biennial, no risk factors). Radiologists recorded screening results and mammographic density (<75%; ≥75%) according to the BI-RADS 4th edition [27] when reviewing mammogram findings and were aware of all previous imaging and clinical history, including family history and other risk factors, prior to interpreting mammograms. Mammograms resulting in a call back for further work-up were considered abnormal. Assessment procedures included breast imaging with or without breast biopsy and final outcomes for each procedure coded as benign or breast cancer. A true negative was defined as a normal mammogram at the index screen date and a false positive was defined as an abnormal mammogram that had a benign assessment at the index screen date.

### 2.4. Selection and Prognostic Characteristics of Breast Cancers

Breast cancers detected within 12 months after the index abnormal screen mammogram episode (abnormal mammogram and assessment) were classified as screen-detected. Interval cancers (false negatives) included those diagnosed before the next screening examination after a normal or benign index screen episode (normal mammogram or abnormal mammogram that had a benign assessment) within one year for annual and two years for biennial. Interval cancers were identified from record linkage using AutoMatch [28] with the Ontario Cancer Registry (OCR), estimated to be 98% complete for breast cancer [29].

Breast cancer histological classification was obtained from the ICMS and from surgical and pathological reports obtained from the OCR and coded using the International Classification of Diseases for Oncology version 3.0 [30]. Reports were reviewed by a trained abstractor and overseen by a breast pathologist (SJD).

### 2.5. Health Resource Utilization Using Administrative Databases

The OBSP cohorts were linked at the individual level using unique encoded identifiers to Ontario’s provincial health administrative databases held at ICES. For overall and false positive outcomes, the cohort was prospectively followed for a 1-year period from index screen to capture the impact of screening recommendations on healthcare costs, while for breast cancers (interval and screen-detected) a 2-year period from the index screen was used to ensure sufficient follow-up time to capture treatment costs, with censoring on date of death or end of Ontario Health Insurance Plan (OHIP) eligibility. Healthcare utilization in the publicly funded system overall and associated with a false positive mammogram (n = 46,081) or breast cancer diagnosis (n = 6011) and time to last follow-up in days were examined.

Inpatient hospitalizations, outpatient and same-day surgery visits were derived from the Canadian Institute for Health Information–Discharge Abstract Database and Same Day Surgery datasets, respectively. The National Ambulatory Care Reporting System dataset was used to identify emergency department visits, as well as cancer clinic visits. Laboratory billings, non-physician and physician visits were derived from OHIP. Prescription medications dispensed to individuals 65 years and older or on social assistance were found using the Ontario Drug Benefit (ODB) database, while expensive intravenous (IV) chemotherapy treatments fall under the New Drug Funding Program. For home care services, complex and continuing care and long-term care (LTC), the Home Care Database, National Rehabilitation (Reporting) System and Continuing Care Reporting System-LTC datasets were utilized.

### 2.6. Health System Resource Costing Analyses

Health system resource utilization costs per woman were obtained using three costing algorithms, which were updated to reflect costs in 2018 Canadian dollars. The %getcost SAS macro developed at ICES [31] calculated cost results for a wide range of health system encounters including physician billing, hospitalizations and emergency visits. In collaboration with ICES, the %getchemocost macro provides chemotherapy-specific costs using data for prescription and IV medications, patient level activity for systemic therapy services, outpatient cancer clinic visits and cancer specific drug lists (updated January 2018) [32]. The %getradiation macro provides radiation-specific costs using data from physician billing, outpatient visits, financial systems and medical physicists’ salaries [32]. Since there is an overlap in some of the resources measured between the %getcost and %getradiationcost macro, some variables were excluded to avoid cost duplication.

Health system resource-specific costs per woman were calculated by screening recommendation, stratified by age and outcome. Chemotherapy and radiation costs were also examined per breast cancer diagnosis. Age-adjusted mean health resource costs per woman were calculated using a generalized regression model with gamma distribution, while age-adjusted median costs were estimated using a quantile regression model [33]. The 95% confidence intervals (CI) of the age-adjusted costs were obtained using 1000 bootstrap samples (randomly selected with replacement). Age-adjusted costs for the annual screening cohorts were compared with the biennial cohort and the 95% CI were based on the 2.5th and 97.5th percentile of the cost difference from the bootstrap samples. Age-adjusted incremental cost differences were determined by subtracting the mean and median of each annual screening group from that of the biennial (reference) group and 95% CI calculated using the Markov chain marginal bootstrap resampling method. All analyses were performed using SAS version 9.4 [34].

## 3. Results

### 3.1. Demographics

**Figure 1:**
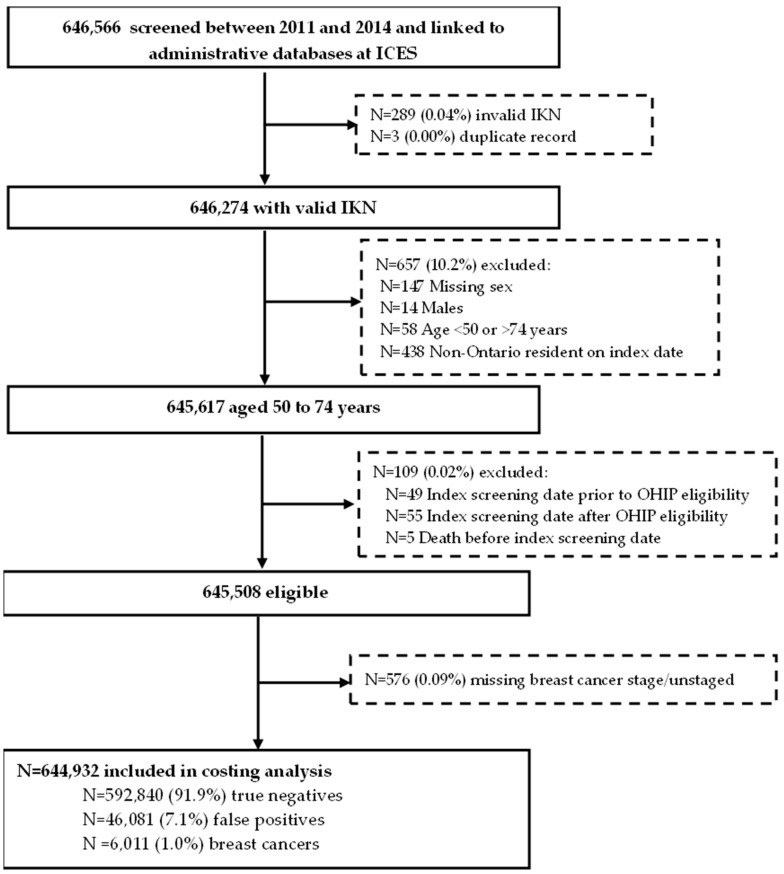
Cohort of eligible women aged 50 to 74 years screened in the Ontario Breast Screening Program between January 2011 and December 2014 with a true negative or false positive mammogram or breast cancer diagnosis at index screen date. Abbreviations: ICES Key Number = IKN; Ontario Health Insurance Plan = OHIP.

Among the 644,932 women included in the final analysis (Figure 1), 592,840 (91.9%) had a true negative, 46,081 (7.1%) had a false positive and 6011 (0.9%) had a breast cancer diagnosis at index screen date. Women screened annually for density ≥ 75% were younger, a higher proportion lived in an urban setting and were in the highest income quintile compared to those screened biennially or annually for family/personal history (Table 1). This risk group also had fewer comorbidities and lower mortality. Follow-up (days) 1 or 2 years after the index screen date was similar across all three screening recommendations.

### 3.2. Costs

Mean healthcare costs overall in the first year after screening were highest among those screened annually for family/personal history (CAD 4685) and lowest for those with density ≥ 75% (CAD 3366) compared to biennially (CAD 3767) (Table 2). The mean incremental cost difference was 24.4% higher per woman (CAD 918; 95% CI: 825 to 1022) for annual screening for family/personal history compared to biennial. By age group, mean costs were lowest for those 50–59 screened annually for density ≥ 75% (CAD 2388; 95% CI: 2299 to 2489), while highest for those 60–74 screened annually for family/personal history (CAD 5425; 95% CI: 5308 to 5557) compared to biennially.

**Figure 2:**
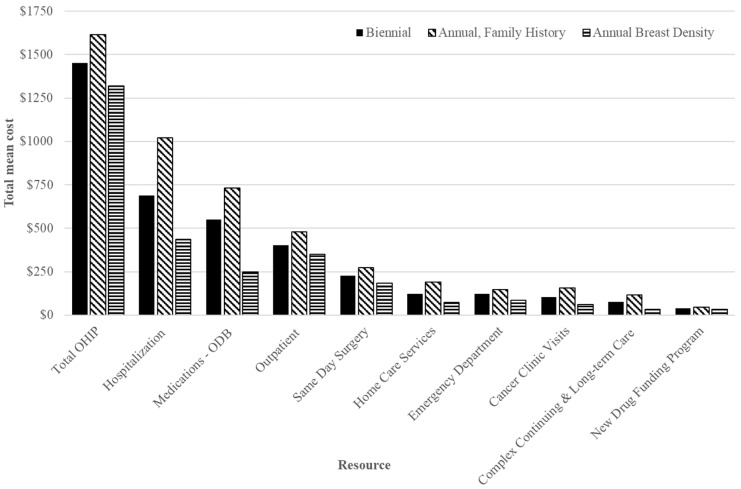

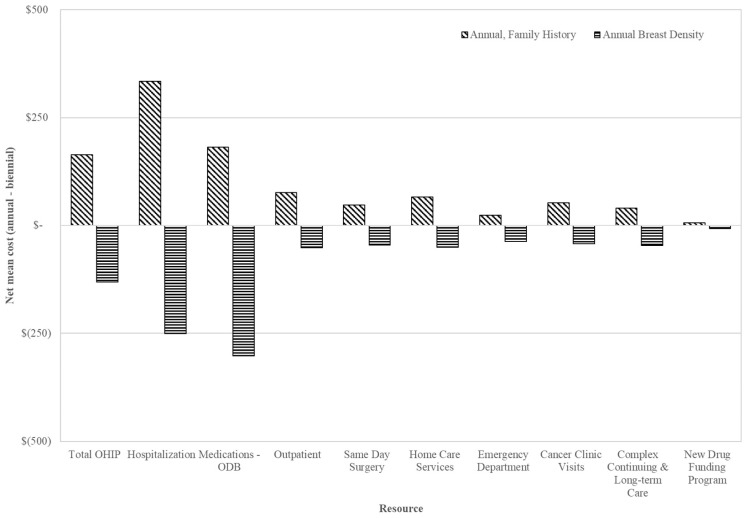
Total mean costs (**top**) and net mean costs (annual minus biennial; (**bottom**)) in 2018 CAD of health specific resources for false positives 1 year after index screen by screening recommendation among those aged 50–74 years. Abbreviations: Ontario Drug Benefit = ODB; Ontario Health Insurance Plan = OHIP; Average exchange rate in 2018: 1.00 USD = 1.2965 CAD.

The highest mean healthcare cost per false positive 1 year after screening was CAD 4605 for those screened annually for family/personal history, while lowest at CAD 3387 among those screened annually for density ≥ 75% and CAD 3847 for those screened biennially (Table 3). The mean incremental cost difference was 12.0% lower (CAD −461; 95% CI: −777 to −114) per false positive for those screened annually for density ≥ 75%, compared to biennially. For annual screening for family/personal history, the mean cost difference per false positive was 19.7% higher than for biennial (CAD 758; 95% CI: 404 to 1118); the greatest cost drivers included inpatient hospitalizations, prescription medications (ODB) and OHIP billings (Figure 2). Among those 50–59, mean costs were lowest for those screened annually for density ≥ 75% (CAD 2258; 95% CI: 2082 to 2466) (Figure S1), while for those 60–74 they were highest for family/personal history (CAD 5383; 95% CI: 4933 to 5857) (Figure S2) compared to biennially.

**Figure 3:**
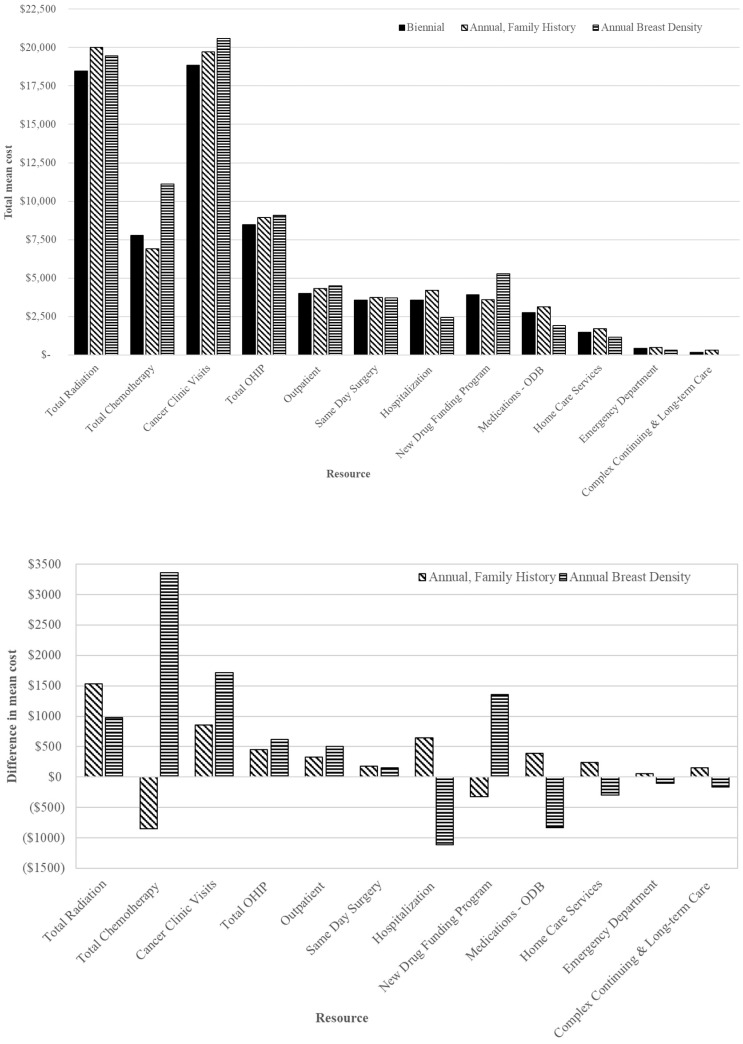
Total mean costs (**top**) and net mean costs (annual minus biennial; (**bottom**)) in 2018 CAD of health specific resources for breast cancers in the first 2-years after index screen by screening recommendation among those aged 50–74 years. Abbreviations: Ontario Drug Benefit = ODB; Ontario Health Insurance Plan = OHIP; Average exchange rate in 2018: 1.00 USD = 1.2965 CAD.

The highest mean cost in the first 2 years after screening per breast cancer was CAD 53,973 among women screened annually for density ≥ 75% compared to CAD 44,831 for those screened annually for family/personal history and CAD 43,738 for biennially (Table 4). The mean incremental cost difference was higher by 23.4% (CAD 10,235; 95% CI: 6141 to 14,329) per breast cancer for those screened annually for density ≥ 75% compared to biennial; the greatest cost drivers included cancer clinic visits and IV medications under the New Drug Funding Program (Figure 3). Among women screened annually for family/personal history, the mean incremental cost difference per breast cancer was only slightly higher (2.5%) by CAD 1093 (95% CI: −1337 to $3760) compared to biennially. Among those 50–59 screened annually, the mean incremental cost difference was highest per breast cancer for density ≥ 75% by 11.4% (CAD 5487; 95% CI: 840 to 10,976) compared to biennially, with the main cost drivers being cancer clinic visits and IV medications (Figure S3). Conversely, among those 60–74, the mean incremental cost difference was greater for those screened annually for family/personal history by 5.3% (CAD 2432; 95% CI: −470 to 5489) compared to biennially; the greatest cost driver was cancer clinic visits (Figure S4).

Total mean chemotherapy costs per breast cancer were highest for those screened annually for density ≥ 75% (CAD 11,131) and lowest for those screened annually for family/personal history (CAD 6916) compared to biennially (CAD 7770), with the greatest costs among those 50–59 with density ≥ 75% (CAD 17,199) (Table 5 and Figure 3). In contrast, mean radiation costs were similar between those screened annually for family/personal history (CAD 20,010) or for density (CAD 19,449), while lowest for those screened biennially (CAD 18,474); a similar pattern was observed among those aged 50–59 and 60–74.

## 4. Discussion

This study compared health resource utilization costs and incremental cost differences (2018 CAD) in the first 1 or 2 years after screening by recommendation among 644,932 women aged 50–74 in the OBSP. Mean 1-year healthcare costs per woman were highest for those aged 60–74, particularly annual screening for family/personal history (CAD 4685). Similarly, per false positive the highest cost 1 year after screening was CAD 5383 for those aged 60–74 screened annually for family/personal history. Mean 2-year healthcare costs per breast cancer were highest at CAD 53,731 for those aged 50–59 screened annually for density ≥ 75%.

Women screened annually for family/personal history had higher mean costs per false positive compared to biennially, and costs were highest for those 60–74. The cost drivers for false positives among those with family/personal history were primarily inpatient hospitalizations, physician billings and prescription medications and may in part also reflect their higher non-malignancy biopsy rate compared to biennial [21]. Interestingly, although specificity was lower for annual screenings for density ≥ 75%, and abnormal recall and non-malignant biopsy rates were higher [21], the mean cost per false positive was lower compared to biennial. These women were younger and had fewer comorbidities than those screened biennially or annually for family/personal history. These factors may have offset costs associated with false positives for those with density ≥ 75%.

An earlier study reported that the mean cost per breast cancer case from a public payer perspective in the first 2 years after diagnosis was CAD 41,686 [23]. These costs are comparable to our study using 2018 Canadian dollars for women screened annually for family/personal history (CAD 44,831) and biennially (CAD 43,738). Cost differences for breast cancers were also higher in both annual screening groups compared to biennial irrespective of age group, but they were lower for women ages 60 to 74 compared to those 50 to 59.

Women screened annually for density ≥ 75% had higher mean costs per breast cancer in the first 2 years after screening, with cancer clinic visits and medications being the primary cost drivers. Within this risk group, those 50–59 had the greatest cost per breast cancer and their cancer medication and chemotherapy-specific costs were also higher, which might be explained by their increased risk of invasive interval versus screen-detected cancers [22]. Since interval cancers are more likely to be diagnosed at a later stage [3,4,5], the cost associated with treatment might be expected to be higher. An earlier study found that higher healthcare costs were associated with increasing breast cancer stage [23]. Although women screened annually for family/personal history had slightly higher costs per breast cancer, they were more similar to those screened biennially than annually for density. This might reflect the higher sensitivity and lower risk of interval or higher stage invasive cancers in those with family/personal history [21,22].

The strengths of this study include the use of large concurrent cohorts within an organized screening program and examination of healthcare costs by screening recommendation based on risk factors. The study limitations include family/personal history based on self-reported data, although the accuracy of reporting breast cancer in first-degree relatives has generally been found to be high [35]. Ontario data sources were collected for administrative purposes; they might therefore not contain all variables of interest with respect to the medical management of breast cancer screening and diagnosis. Total costs for false positives considered all health system resources 1 year after screening, which may or may not be attributable to screening recommendation or outcome and may represent management of other comorbidities. Lastly, it is evident that analyzing data in the 2 years after screening might not accurately identify all costs and utilization of breast cancer management, because for many patients, treatment and survival can extend beyond these years.

Among breast screening programs, uncertainties exist regarding effective recommendations for women at increased risk. In the OBSP, annual breast screening based on family/personal history improved cancer detection and lowered the risk of interval or higher stage invasive cancers [21,22]. However, these benefits were associated with increased costs following a false positive result and slightly higher costs of breast cancers. For women with density ≥ 75%, the costs per breast cancer were highest, which may reflect the increased risk of invasive interval versus screen-detected cancers, especially in those 50–59 [22]. However, the higher risk of false positive screens in the annual density group did not increase costs. Understanding that the incremental costs of annual compared to biennial screening may balance out by age and outcome can assist decision-making regarding use of limited healthcare resources. Irrespective of the associated costs, women at increased risk should be advised of the benefits and harms of annual screening based on their risk factors.

## Figures and Tables

**Table 1:** Baseline characteristics for women with a true negative or false positive mammogram or breast cancer diagnosis at index screen date by screening recommendation.

	True Negatives (N = 592,840)			False Positives (N = 46,081)			Breast Cancers (N = 6011)		
Baseline Characteristics	Biennial N = 482,748	Annual, Family/Personal History N = 62,674	Annual, Density ≥ 75% N = 47,418	Biennial N = 38,019	Annual, Family/Personal History N = 4106	Annual, Density ≥ 75% N = 3956	Biennial N = 4734	Annual, Family/Personal History N = 829	Annual, Density ≥ 75% N = 448
Age (y) at index screen	62.2 (6.3)	63.2 (6.6)	58.8 (6.2)	61.7 (6.3)	62.7 (6.7)	58.2 (6.1)	63.7 (6.0)	64.4 (5.9)	59.9 (6.6)
Mean (SD)	62.0	63.0	57.0	61.0	62.0	56.0	64.0	65.0	59.0
Median (IQR)	(57.0–67.0)	(58.0–69.0)	(54.0–63.0)	(56.0–67.0)	(57.0–68.0)	(53.0-62.0)	(59.0–69.0)	(60.0–69.0)	(54.0–65.0)
Residence location (n,%)									
Urban	413,570 (85.7)	52,042 (83.0)	43,310 (91.3)	32,643 (85.9)	3482 (84.8)	3598 (91.0)	4034 (85.2)	701 (84.6)	404 (90.2)
Rural	69,000 (14.3)	10,598 (16.9)	4077 (8.6)	5368 (14.1)	621 (15.1)	355 (9.0)	699 (14.8)	128 (15.4)	44 (9.8)
Missing	178 (0.0)	34 (0.1)	31 (0.1)	8 (0.0)	≤5 (0.1)	≤5 (0.1)	≤5 (0.0)	≤5 (0.0)	≤5 (0.0)
Income Quintile (n,%)									
Q1 (lowest)	74,737 (15.5)	10,530 (16.8)	6338 (13.4)	5748 (15.1)	713 (17.4)	495 (12.5)	728 (15.4)	155 (18.7)	53 (11.8)
Q2	92,588 (19.2)	12,306 (19.6)	8340 (17.6)	7170 (18.9)	825 (20.1)	638 (16.1)	882 (18.6)	160 (19.3)	85 (19.0)
Q3	97,883 (20.3)	12,819 (20.5)	9025 (19.0)	7578 (19.9)	809 (19.7)	810 (20.5)	981 (20.7)	172 (20.7)	77 (17.2)
Q4	103,787 (21.5)	12,942 (20.6)	10,356 (21.8)	8152 (21.4)	832 (20.3)	871 (22.0)	1000 (21.1)	170 (20.5)	96 (21.4)
Q5 (highest)	112,910 (23.4)	13,996 (22.3)	13,283 (28.0)	9297 (24.5)	923 (22.5)	1134 (28.7)	1139 (24.1)	172 (20.7)	137 (30.6)
Missing	843 (0.2)	81 (0.1)	76 (0.2)	74 (0.2)	≤5 (0.1)	8 (0.2)	≤5 (0.1)	≤5 (0.0)	≤5 (0.0)
Charlson Comorbidity Index (n,%)									
No hospitalization	333,590 (69.1)	40,368 (64.4)	35,605 (75.1)	26,215 (69.0)	2591 (63.1)	2946 (74.5)	3118 (65.9)	527 (63.6)	310 (69.2)
0	127,065 (26.3)	18,449 (29.4)	10,889 (23.0)	10,038 (26.4)	1272 (31.0)	924 (23.4)	1384 (29.2)	245 (29.6)	125 (27.9)
1	14,160 (2.9)	2346 (3.7)	565 (1.2)	1105 (2.9)	139 (3.4)	49 (1.2)	164 (3.5)	39 (4.7)	9 (2.0)
2+	7933 (1.6)	1511 (2.4)	359 (0.8)	661 (1.7)	104 (2.5)	37 (0.9)	68 (1.4)	18 (2.2)	≤5 (0.9)
End of follow-up reason (n,%) ^a^									
Death	1657 (0.3)	488 (0.8)	123 (0.3)	125 (0.3)	35 (0.9)	11 (0.3)	59 (1.2)	16 (1.9)	≤5 (0.1)
1-year after index screen date	480,813 (99.6)	62,120 (99.1)	47,251 (99.6)	37,868 (99.6)	4066 (99.0)	3943 (99.7)	--	--	--
2 years after index screen date	--	--	--	--	--	--	4671 (98.7)	812 (97.9)	447 (99.8)
End of OHIP eligibility	278 (0.1)	66 (0.1)	26 (0.1)	26 (0.1)	≤5 (0.1)	≤5 (0.1)	≤5 (0.1)	≤5 (0.1)	≤5 (0.1)
Follow-up (days) ^b^	364.6 (12.1)	363.9 (17.1)	364.7 (11.3)	364.6 (11.3)	363.8 (17.0)	364.8 (9.2)	726.7 (39.4)	725.6 (44.7)	729.3 (25.2)
Mean (SD)	365.0	365.0	365.0	365.0	365.0	365.0	731.0	731.0	731.0
Median (IQR)	(365.0–365.0)	(365.0–365.0)	(365.0–365.0)	(365.0–365.0)	(365.0–365.0)	(365.0–365.0)	(731.0–731.0)	(731.0–731.0)	(731.0–731.0)

Abbreviations: Interquartile Range (IQR); standard deviation (SD); Ontario Health Insurance Plan (OHIP). ^a^ End of follow-up reason for health resource costing is 1 year from index screen date for true negatives and false positives; for breast cancers it is 2 years from index screen date. ^b^ Follow-up (days) for health resource costing is 1 year from index screen date for true negatives and false positives; for breast cancers it is 2 years from index screen date.

**Table 2:** Age-adjusted mean and median total health resource costs (2018 CAD) ^a^ and incremental cost differences 1 year after screening by age and screening recommendation (n = 644,932).

Total Health Resource Costs	Biennial	Annual, Family or Personal History	Annual, Mammographic Density ≥ 75%
Age (y) 50–74	N = 525,501	N = 67,609	N = 51,822
Mean (95% CI) ^b^	3767 (3740 to 3794)	4685 (4593 to 4783)	3366 (3271 to 3449)
Incremental Cost (95% CI)	Reference	918 (825 to 1022)	−401 (−500 to −311)
Median (95% CI) ^c^	1260 (1251 to 1269)	1455 (1438 to 1471)	1327 (1303 to 1344)
Incremental Cost (95% CI)	Reference	195 (177 to 213)	66 (41 to 85)
Age (y) 50–59	N = 203,604	N = 22,164	N = 31,800
Mean (95% CI) ^b^	2661 (2626 to 2696)	3624 (3472 to 3795)	2388 (2299 to 2489)
Incremental Cost (95% CI)	Reference	964 (811 to 1133)	−273 (−365 to −166)
Median (95% CI) ^c^	901 (892 to 909)	1035 (1013 to 1059)	826 (813 to 836)
Incremental Cost (95% CI)	Reference	134 (110 to 160)	−75 (−90 to −60)
Age (y) 60–74	N = 321,897	N = 45,364	N = 20,022
Mean (95% CI) ^b^	4499 (4460 to 4536)	5425 (5308 to 5557)	3968 (3842 to 4103)
Incremental Cost (95% CI)	Reference	926 (802 to 1072)	−531 (−669 to −393)
Median (95% CI) ^c^	1649 (1638 to 1661)	1828 (1780 to 1901)	1534 (1502 to 1566)
Incremental Cost (95% CI)	Reference	179 (130 to 254)	−115 (−149 to −81)

Abbreviations: Confidence interval (CI); Interquartile Range (IQR); standard deviation (SD). ^a^ Average exchange rate in 2018: 1.00 USD = 1.2965 CAD. ^b^ Age-adjusted mean cost estimated using generalized regression; age-adjusted incremental cost difference estimated using bootstrapping with biennial screening recommendation as reference group. ^c^ Age adjusted median cost estimated using quantile regression; age-adjusted median incremental cost difference estimated using bootstrapping with biennial screening recommendation as reference group.

**Table 3:** Age-adjusted mean and median health resource costs (2018 CAD) ^a^ and incremental cost differences 1 year after screening for false positives (n = 46,081) by age and screening recommendation.

Health Resource Costs	Biennial	Annual, Family or Personal History	Annual, Mammographic Density ≥ 75%
Age (y) 50–74	N = 38,019	N = 4106	N = 3956
Mean (95% CI) ^b^	3847 (3759 to 3942)	4605 (4267 to 4947)	3387 (3101 to 3733)
Incremental Cost (95% CI)	Reference	758 (404 to 1188)	−461 (−777 to −114)
Median (95% CI) c	1511 (1488 to 1533)	1685 (1530 to 1759)	1742 (1553 to 1818)
Incremental Cost (95% CI)	Reference	174 (19 to 249)	232 (42 to 307)
Age (y) 50–59	N = 15,798	N = 1484	N = 2592
Mean (95% CI) ^b^	2771 (2656 to 2877)	3427 (2918 to 3877)	2258 (2082 to 2466)
Incremental Cost (95% CI)	Reference	657 (163 to 1130)	−513 (−725 to −278)
Median (95% CI) ^c^	1108 (1085 to 1131)	1285 (1212 to 1383)	1057 (997 to 1114)
Incremental Cost (95% CI)	Reference	177 (101 to 280)	−51 (−119 to 12)
Age (y) 60–74	N = 22,221	N = 2622	N = 1364
Mean (95% CI) ^b^	4558 (4418 to 4699)	5383 (4933 to 5857)	4245 (3663 to 5081)
Incremental Cost (95% CI)	Reference	824 (338 to 1323)	−313 (−912 to 522)
Median (95% CI) ^c^	1862 (1826 to 1901)	1761 (1733 to 1795)	1830 (1742 to 2006)
Incremental Cost (95% CI)	Reference	145 (43 to 373)	−32 (−132 to 150)

Abbreviations: Confidence interval (CI); Interquartile Range (IQR); standard deviation (SD). ^a^ Average exchange rate in 2018: 1.00 USD = 1.2965 CAD; ^b^ age-adjusted mean cost estimated using generalized regression; age-adjusted incremental cost difference estimated using bootstrapping with biennial screening recommendation as reference group; ^c^ age adjusted median cost estimated using quantile regression; age-adjusted median incremental cost difference estimated using bootstrapping with biennial screening recommendation as reference group.

**Table 4:** Age-adjusted mean and median health resource costs (2018 CAD) ^a^ and incremental cost differences in the first 2 years after screening for breast cancers (screen-detected and interval) (n = 6011) by age and screening recommendation.

Health Resource Costs	Biennial	Annual, Family or Personal History	Annual, Mammographic Density ≥ 75%
Age (y) 50–74	N = 4734	N = 829	N = 448
Mean (95% CI) ^b^	43,738 (42,749 to 44,751)	44,831 (42,588 to 47,449)	53,973 (49,935 to 57,845)
Incremental Cost (95% CI)	Reference	1093 (−1337 to 3760)	10,235 (6141 to 14,329)
Median (95% CI) ^c^	30,702 (30,115 to 31,317)	31,091 (30,193 to 33,564)	43,357 (40,070 to 47,279)
Incremental Cost (95% CI)	Reference	389 (−678 to 2917)	12,655 (9337 to 16,421)
Age (y) 50–59	N = 1331	N = 183	N = 241
Mean (95% CI) ^b^	48,244 (46,409 to 50,167)	51,792 (47,129 to 56,860)	53,731 (49,537 to 59,032)
Incremental Cost (95% CI)	Reference	3548 (−1653 to 9046)	5487 (840 to 10,976)
Median (95% CI) ^c^	37,868 (36,293 to 39,140)	41,832 (37,928 to 47,017)	41,827 (37,410 to 45,244)
Incremental Cost (95% CI)	Reference	3965 (−222 to 9301)	3959 (−708 to 7974)
Age (y) 60–74	N = 3403	N = 646	N = 207
Mean (95% CI) ^b^	45,555 (44,351 to 46,771)	47,987 (45,403 to 50,959)	45,878 (41,410 to 51,167)
Incremental Cost (95% CI)	Reference	2432 (−470 to 5489)	323 (−4134 to 5818)
Median (95% CI) ^c^	33,609 (32,563 to 35,236)	35,657 (33,459 to 38,406)	35,868 (32,661 to 40,615)
Incremental Cost (95% CI)	Reference	2048 (−665 to 4981)	2259 (−1420 to 7017)

Abbreviations: Confidence interval (CI); Interquartile Range (IQR); standard deviation (SD). ^a^ Average exchange rate in 2018: 1.00 USD = 1.2965 CAD; ^b^ age-adjusted mean cost estimated using generalized regression; age-adjusted incremental cost difference estimated using bootstrapping with biennial screening recommendation as reference group; ^c^ age-adjusted median cost estimated using quantile regression; age-adjusted median incremental cost difference estimated using bootstrapping with biennial screening recommendation as reference group.

**Table 5:** Mean and median health resource costs (2018 CAD) ^a^ and incremental cost differences in the first 2 years after screening for breast cancers (screen-detected and interval) (n = 6011) by age and screening recommendation.

**Cancer Medication and Chemotherapy Costs**	**Biennial**	**Annual, Family or** **Personal History**	**Annual, Mammographic density ≥ 75%**
Age (y) 50–74	N = 4734	N = 829	N = 448
Mean (±SD)	7770 ± 17,199	6916 ± 16,791	11,131 ± 21,021
Median (IQR)	1018 (291 to 3787)	938 (263 to 3668)	1547 (401 to 8145)
Incremental Cost (95% CI)	Reference	−80 (−208 to 48)	516 (−40 to 1072)
Age (y) 50–59	N = 1331	N = 183	N = 241
Mean (±SD)	14,285 ± 21,556	10,937 ± 20,374	17,199 ± 23,212
Median (IQR)	3054 (968 to 16,341)	2571 (1028 to 7737)	3304 (916 to 36,825)
Incremental Cost (95% CI)	Reference	−481 (−1429 to 467)	233 (−2051 to 2517)
Age (y) 60–74	N = 3403	N = 646	N = 207
Mean (±SD)	6364 ± 15,759	6200 ± 15,985	6884 ± 18,246
Median (IQR)	896 (241 to 2731)	837 (225 to 3150)	1045 (198 to 3128)
Incremental Cost (95% CI)	Reference	−57 (−157 to 43)	129 (−100 to 358)
**Radiation Costs**	**Biennial**	**Annual, Family or** **Personal History**	**Annual, Mammographic density ≥ 75%**
Age (y) 50–74	N = 4734	N = 829	N = 448
Mean (±SD)	18,474 ± 10,337	20,010 ± 10,882	19,449 ± 11,096
Median (IQR)	19,234 (14,410 to 24,106)	20,549 (15,262 to 26,144)	20,935 (15,047 to 25,332)
Incremental Cost (95% CI)	Reference	1315 (520 to 2111)	1701 (667 to 2735)
Age (y) 50–59	N = 1331	N = 183	N = 241
Mean (±SD)	19,180 ± 10,403	20,629 ± 9675	20,369 ± 10,763
Median (IQR)	19,463 (14,881 to 25,129)	21,352 (16,336 to 25,802)	21,656 (15,733 to 25,713)
Incremental Cost (95% CI)	Reference	1888 (342 to 3434)	2171 (694 to 3648)
Age (y) 60–74	N = 3403	N = 646	N = 207
Mean (±SD)	18,194 ± 10,299	19,829 ± 11,214	18,308 ± 11,423
Median (IQR)	19,027 (14,297 to 23,632)	20,348 (14,830 to 26,150)	20,000 (14,246 to 23,363)
Incremental Cost (95% CI)	Reference	1308 (168 to 2449)	961 (−1026 to 2948)

Abbreviations: Confidence interval (CI); Interquartile Range (IQR); standard deviation (SD). ^a^ Average exchange rate in 2018: 1.00 USD = 1.2965 CAD.

## Data Availability

Parts of the material underlying this article are based on data and information provided by Ontario Health (Cancer Care Ontario) and CIHI. Ontario Health is prohibited from making the data used in this research publicly accessible if it includes potentially identifiable personal health information and/or personal information as defined in Ontario law, specifically the Personal Health Information Protection Act (PHIPA) and the Freedom of Information and Protection of Privacy Act (FIPPA). Upon request, data de-identified to a level suitable for public release may be provided.

## Supplementary Materials

The following supporting information can be downloaded at: https://www.mdpi.com/article/10.3390/curroncol30090620/s1, Figure S1: Total mean costs and net mean costs in 2018 CAD of health specific resources for false positives 1-year after index screen by screening recommendation among those aged 50–59 years; Figure S2: Total mean costs and net mean costs in 2018 CAD of health specific resources for false positives 1-year after index screen by screening recommendation among those aged 60–74 years; Figure S3: Total mean costs and net mean costs in 2018 CAD of health specific resources for breast cancers 2-years after index screen by screening recommendation among those aged 50–59 years; Figure S4: Total mean costs and net mean costs in 2018 CAD of health specific resources for breast cancers 2-years after index screen by screening recommendation among those aged 60–74 years.
