# Supplementary material for: Healthcare and Cancer Treatment Costs of Breast Screening Outcomes among Higher than Average Risk Women
Source: Curr Oncol. 2023 Sep 18;30(9):8550–62. doi: 10.3390/curroncol30090620 (PMC10529052; doi:10.3390/curroncol30090620)

Figure S1. Total mean costs (**top**) and net mean cost (annual minus biennial; (**bottom**)) in 2018 CAD of health specific resources for false positives 1 year after index screen by screening recommendation among those aged 50-59 years. Abbreviations: Ontario Drug Benefit = ODB; Ontario Health Insurance Plan = OHIP; Average exchange rate in 2018: 1.00 USD = 1.2965 CAD.

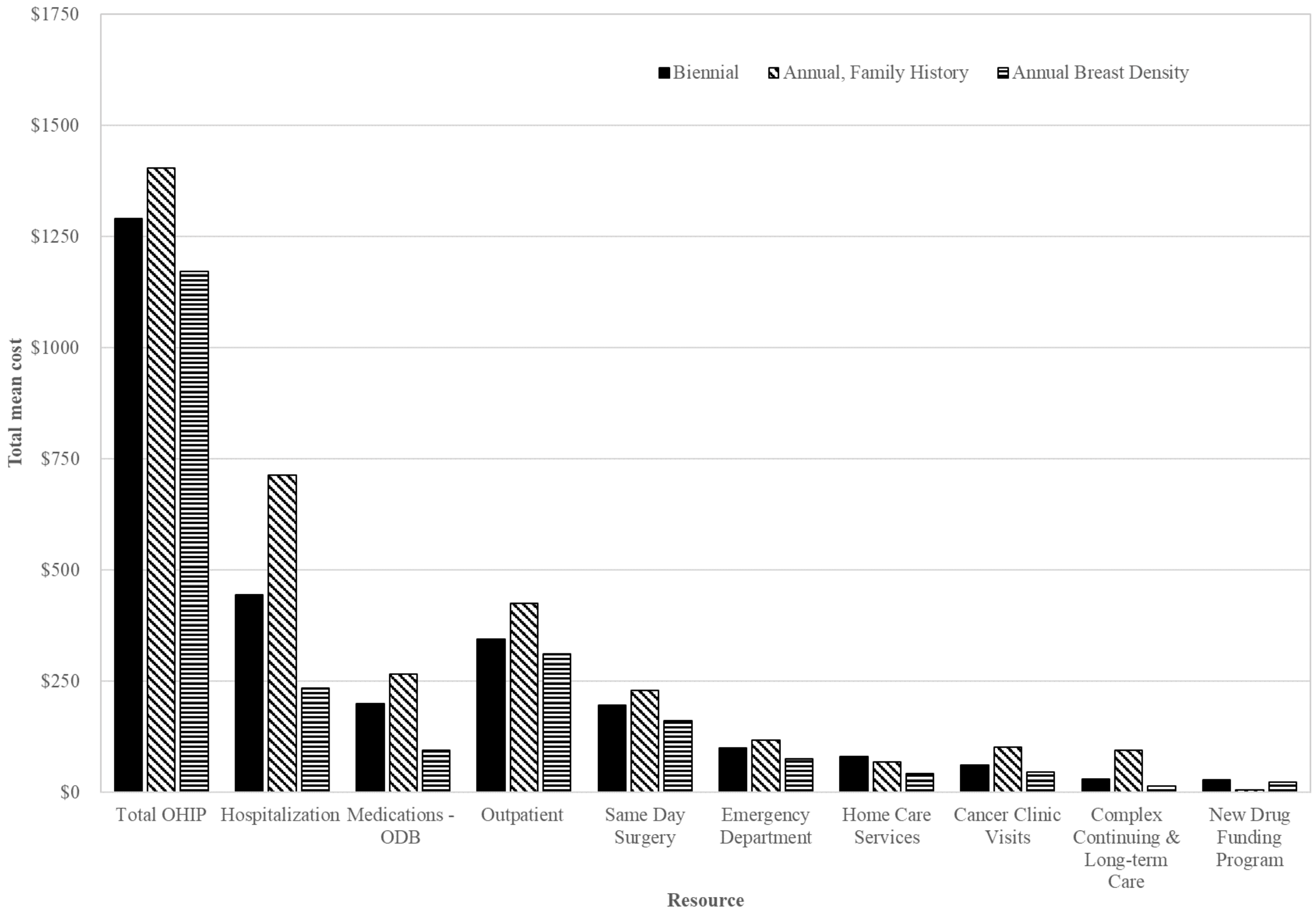

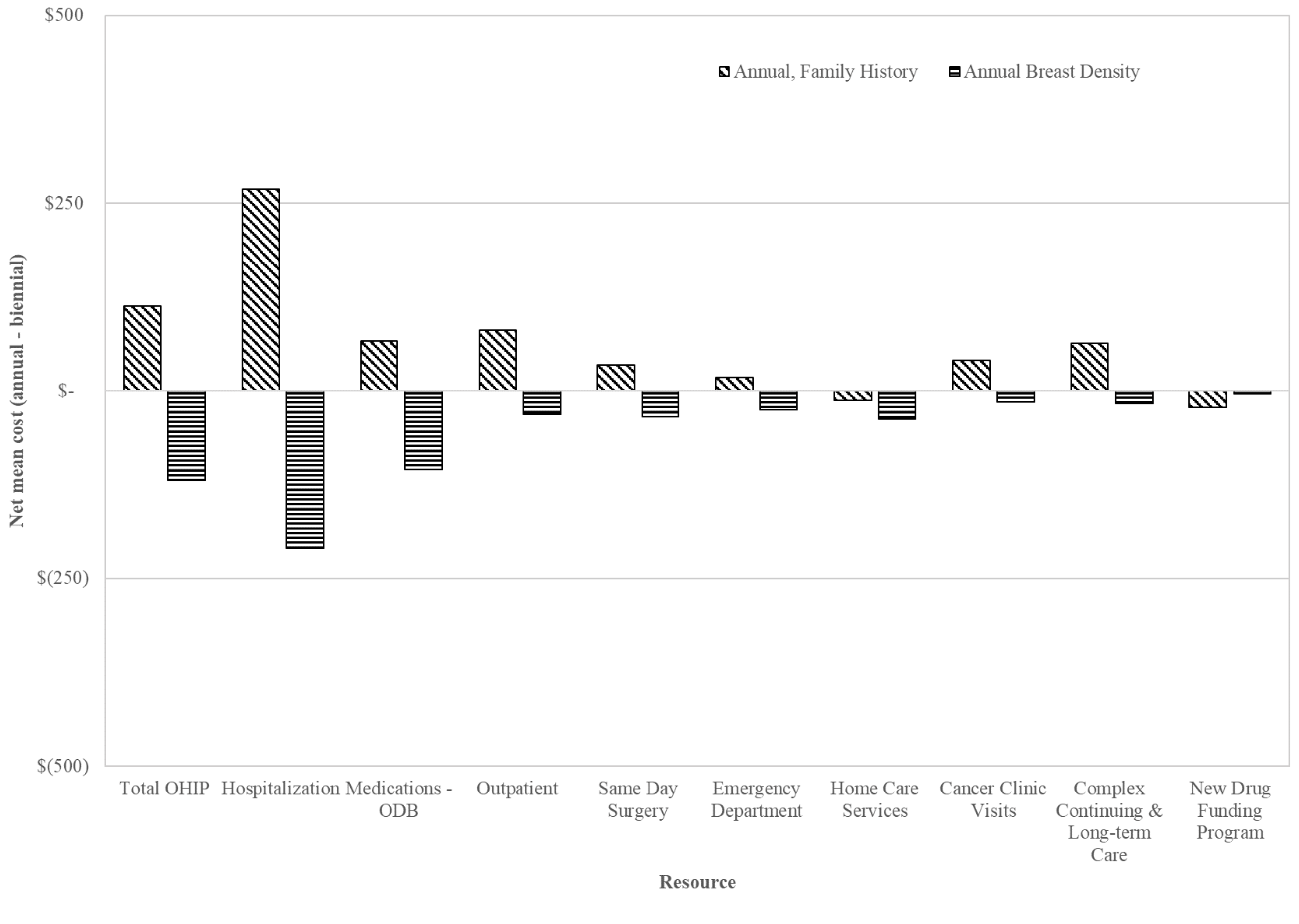

Figure S2. Total mean costs (**top**) and net mean cost (annual minus biennial; (**bottom**)) in 2018 CAD of health specific resources for false positives 1 year after index screen by screening recommendation among those aged 60-74 years. Abbreviations: Ontario Drug Benefit = ODB; Ontario Health Insurance Plan = OHIP; Average exchange rate in 2018: 1.00 USD = 1.2965 CAD.

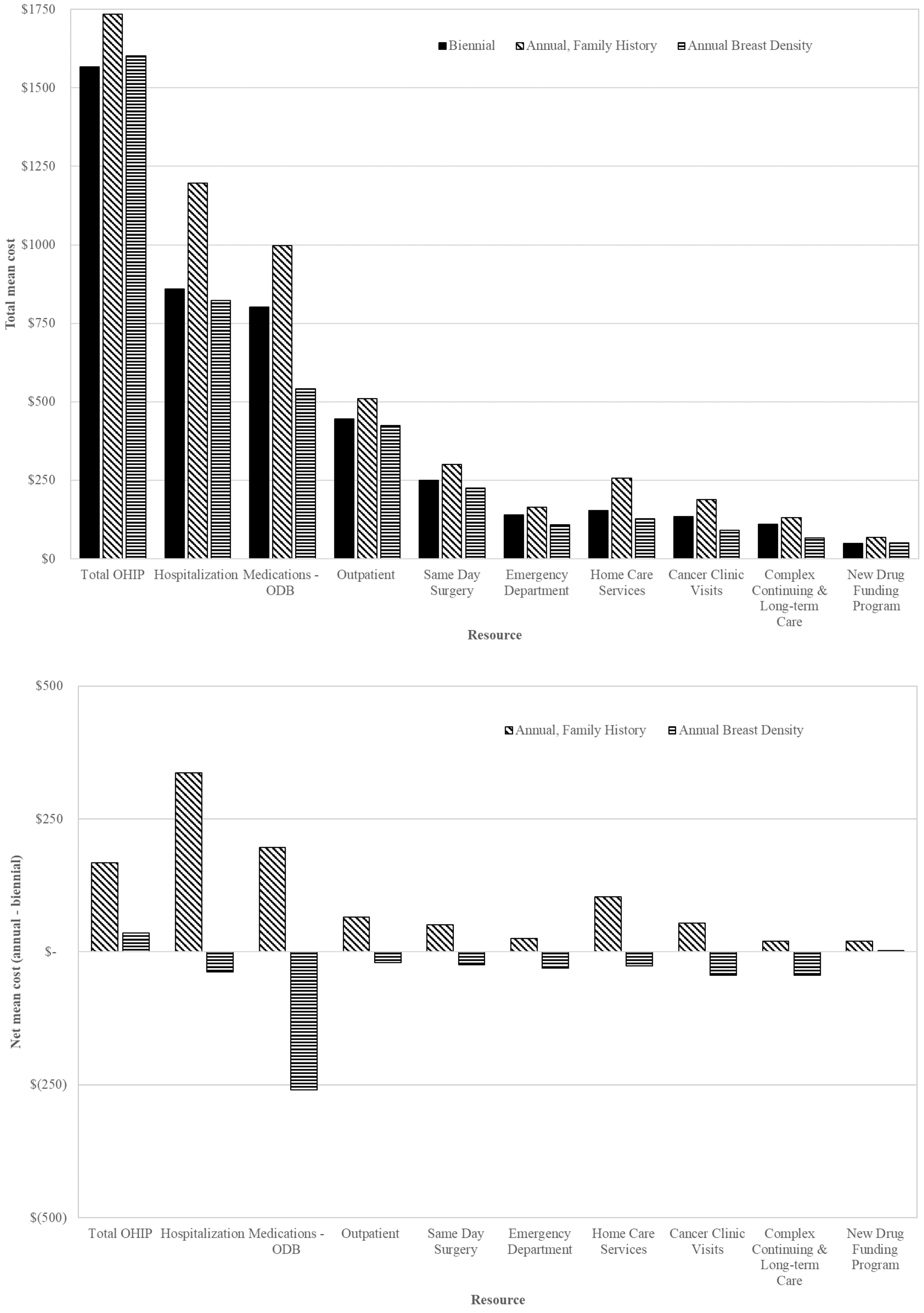

Figure S3. Total mean costs (**top**) and net mean cost (annual minus biennial; (**bottom**)) in 2018 CAD of treatment and health specific resources for breast cancers 2-years after index screen by screening recommendation among those aged 50-59 years. Abbreviations: Ontario Drug Benefit = ODB; Ontario Health Insurance Plan = OHIP; Average exchange rate in 2018: 1.00 USD = 1.2965 CAD.

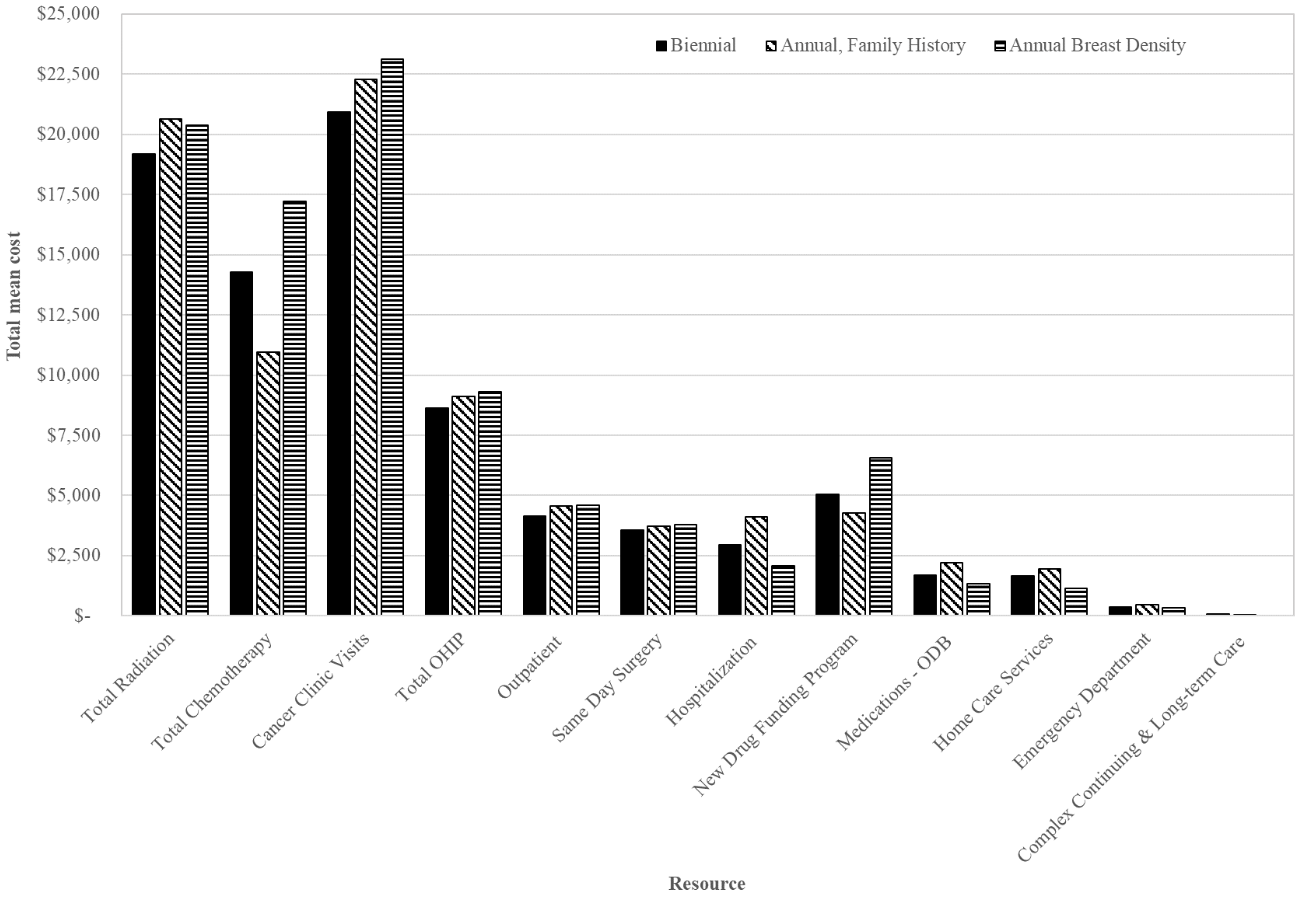

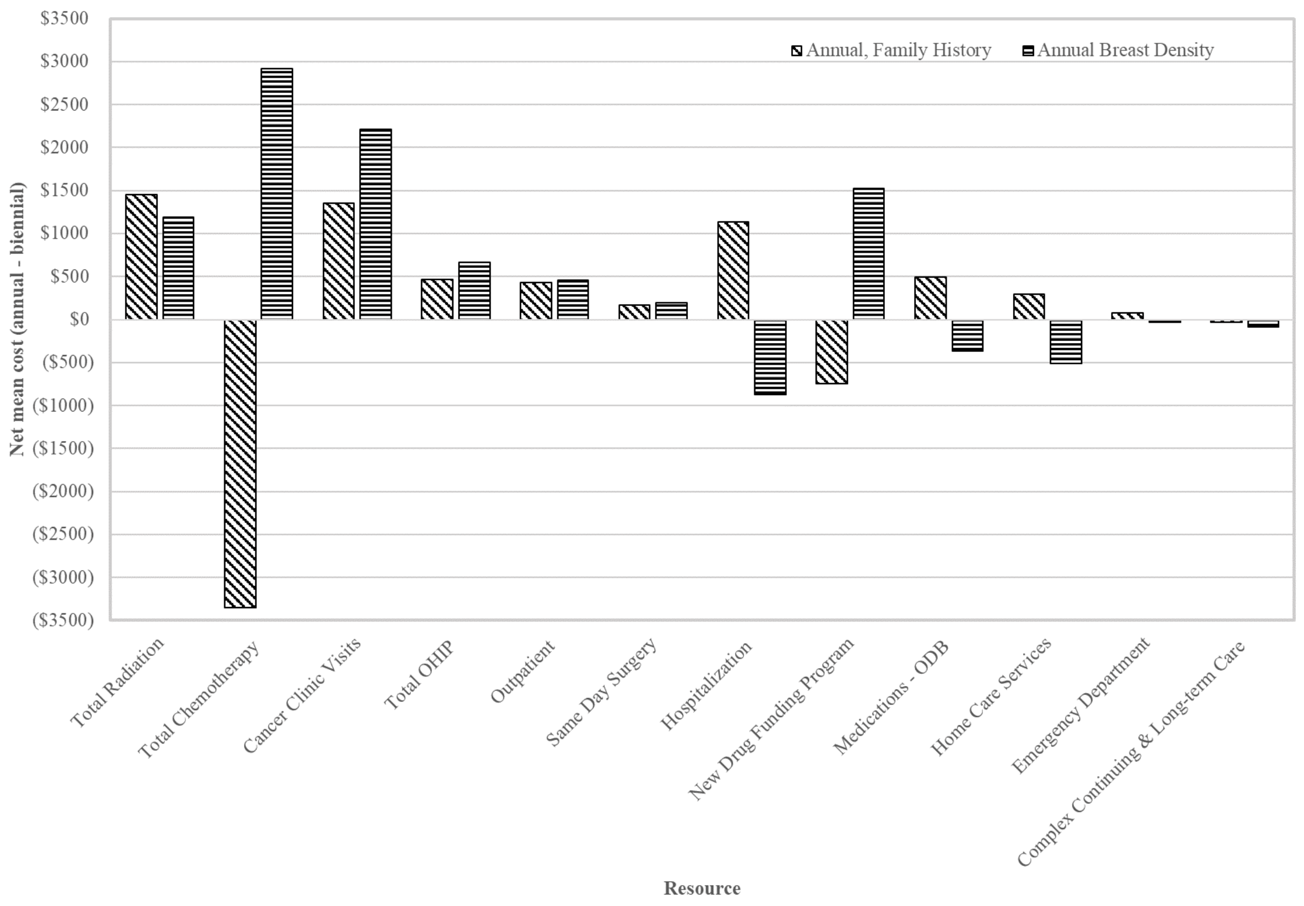

Figure S4. Total mean costs (**top**) and net mean cost (annual minus biennial; (**bottom**)) in 2018 CAD of treatment and health specific resources for breast cancers 2-years after index screen by screening recommendation among those aged 60-74 years. Abbreviations: Ontario Drug Benefit = ODB; Ontario Health Insurance Plan = OHIP; Average exchange rate in 2018: 1.00 USD = 1.2965 CAD.

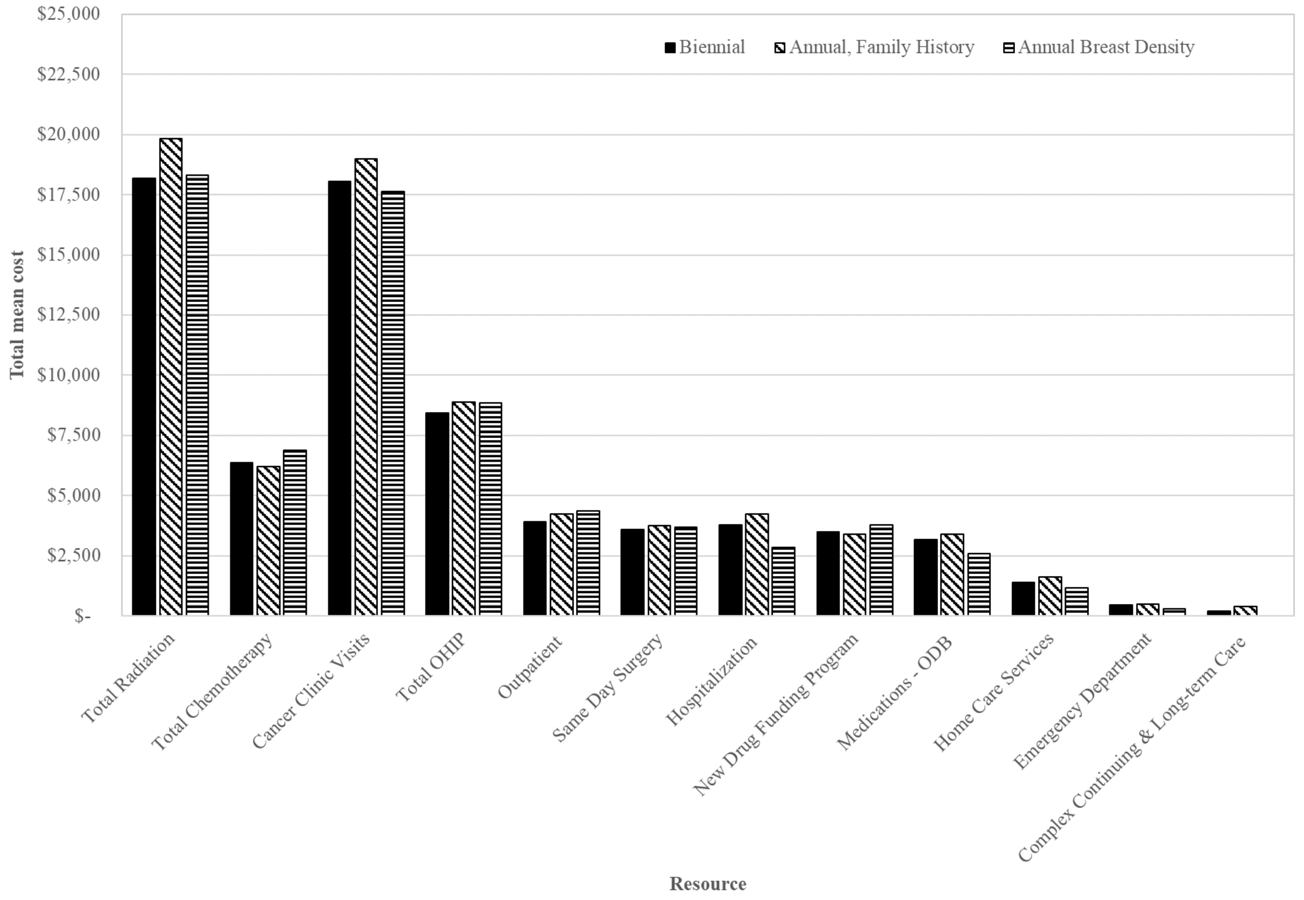

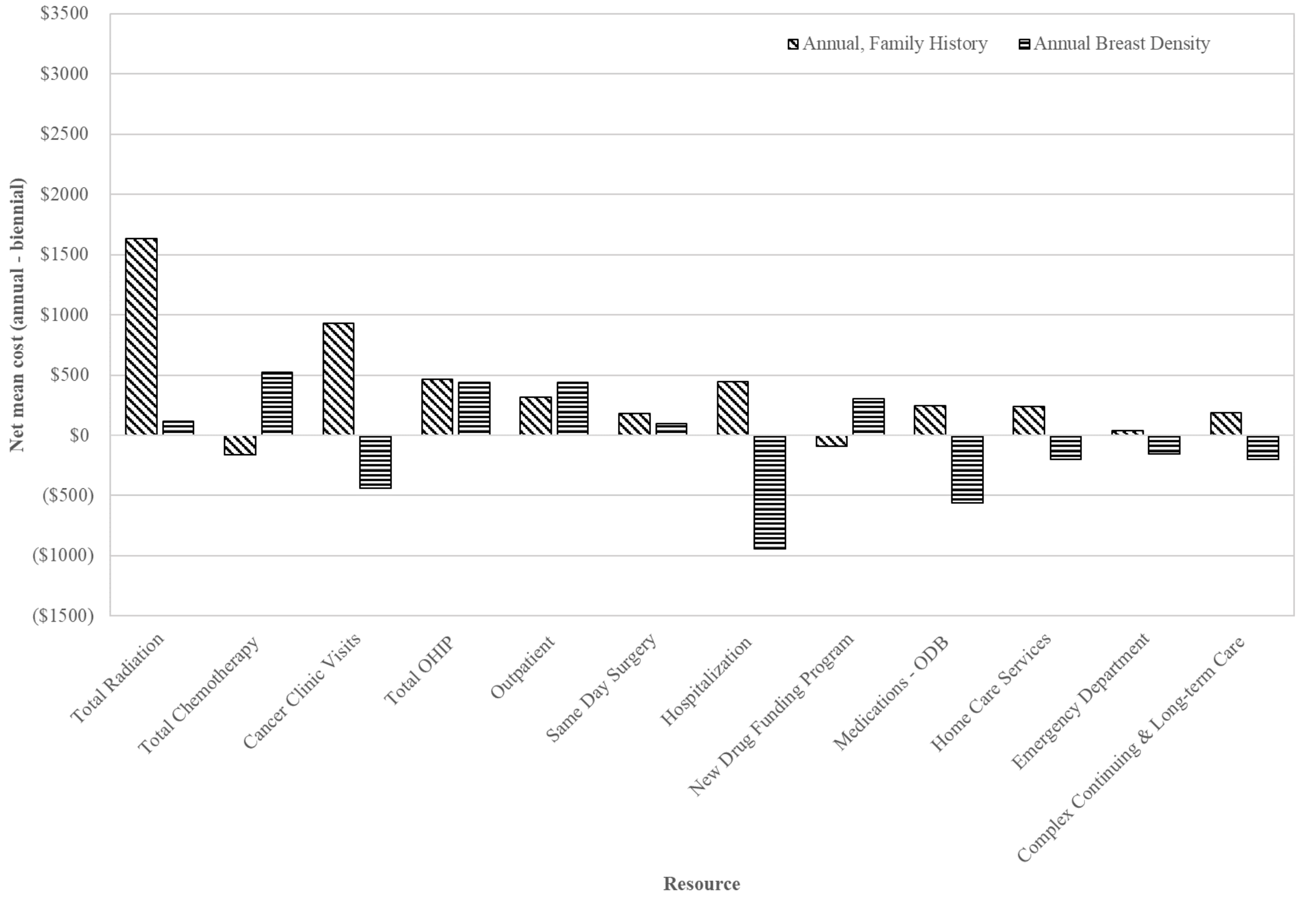

Supplement: Supplementary file 1 [file curroncol-30-00620-s001.zip › curroncol-2562614-supplementary.pdf]
